# Another Food Fad

**Published:** 1916-04-08

**Authors:** 


### ANOTHER FOOD FAD.

The world is never long without some new and confident discovery by which health may be secured and disease banished. Undeterred by a lengthy list of past failures and abandoned hopes, new philanthropists ever appear with the announcement that at last truth has been attained and that the anxieties of the human race may be abated. Sometimes, it is to be feared, the motive power of these gospels is a natural but somewhat vulgar desire to make money, but in other instances, doubtless, philanthropy, not by any means always associated with knowledge, directs the chariot. In either event publicity is a necessity of the position. There are columns which are opened by cash payments, others are reached by sensational achievements, whilst to a third the avenue appears to be provided by social distinction; and there is reason to believe that a combination of these factors in various proportions is not unknown. The great thing, indeed the necessary thing, is to catch the public eye, and there is reasonable certainty that, given appropriate means, this end will be secured—at least for a season. After a time, if history may be trusted, the new discovery will have to make way for a still more modern revelation. Amongst such discoveries artificial agents in the form of various medicines take a prominent place, and if they can be presented as hailing from some land or tribe untainted by the approach of civilisation they are morally sure of a sympathetic and remunerative welcome. But the true pride of place in the popular estimate is reserved for new schemes of diet.

The very latest proposal in this direction is that the really reliable method of securing health and of avoiding or curing disease is to select a diet consisting solely of uncooked fruits and vegetables. Possibly there is an even more perfect system—namely, not to take any food at all. At least this qualification seems to be implied in the announcement of the chief prophet of the movement, who tells us that "the less I ate the more energetic I became," and that after a fortnight's fasting his "energy was simply bounding." Whether during this period he was working as a day labourer or was merely pondering over dietetic problems does not appear. In any event, it seems that at the end of two weeks he had "become very hungry," and, misled by this sensation, he again relapsed into eating, with the result that he at once "felt less well." Then came to him the happy thought which saved him from the pangs of hunger on the one hand and from the sensation of "less well" on the other. He watched, we are told, "the squirrels and rabbits and other animals," and concluded that the way to salvation lay in a diet of fresh uncooked vegetables and fruit. These, in his own expressive phrase, "filled the bill," and now, it is pleasant to learn, he is in "perfect health." Quite properly he refused to keep this great discovery for his own selfish satisfaction, and under his inspiration "it has become the vogue," we learn, among a number of people "well known in Society," to live upon fruit and vegetable salads.

The testimony, however, does not halt with the preacher. One of his converts, a lady who sometimes dines in Downing Street, also gives her testimony, and, though during her five weeks' experience she has occasionally fallen from grace, she tells us that her arthritis is "considerably better"—an announcement, we are sure, everyone will welcome. Equally with her mentor she is not content with mere results, and must needs find a sanction in "Nature." And here it is only justice to say that the pupil outstrips the master. He, it will be remembered, finds his warrant in "squirrels and rabbits," but the lady claims more impressive examples, and refers us with convincing force to the "natural diet which Adam and Eve took in the Garden of Eden." Yet on second thoughts we are not quite sure she has been quite well advised in her argument. On the dietetic habits of the persons whom she mentions we possess only scanty information, but, so far as the story goes, there was in this direction certainly one profound mistake. The "natural" inclination of our first parents towards one variety of fruit can hardly be quoted for the encouragement of their descendants. The practical point, however, is that the arthritis is "considerably better," and on this result we offer our respectful congratulations. If we feel a little hesitation in awarding the full credit of this "cure" to the "natural diet" it is because we remember that not many years ago equally impressive results were chronicled by a sufferer—again a lady of some literary skill—under the influence of a *régime* of an entirely different quality. Here all vegetables, whether cooked or uncooked, were strictly excluded, and the patient was confined to minced beef and boiling water. Yet the benefits claimed were certainly not less than those now attributed to the dietetic habits both of the squirrel and of the parents of the human race. Now comes the difficulty. In spite of the testimonies of the originator and of not a few followers, the minced beef and the boiling water have long since lost their vogue, and the book which proclaimed their virtues, if found at all, may most hopefully be looked for in the "penny box" of the barrows of Farringdon Road. Whether the system was or was not a "cure," it had for a time numerous disciples and no small popularity. Why has it practically ceased to be? Certainly it has not caused the disappearance of "arthritis, rheumatism, etc.," for the victims of these ills are at our doors and flourish even among those, as we now learn, "well known in Society." To-day appears a new "cure" which, so far as it has any principle at all, is in direct opposition to the beef-and water business. If oblivion may attend the one, what guarantee have we that a similar fate does not await the other? In the face of such an experience we must confess that the squirrel does not move us, and while we profess no desire to "reform" his habits we are equally resolved that neither shall he set the pattern for ours.

